# Genome analysis of *Bifidobacterium adolescentis* and investigation of its effects on inflammation and intestinal barrier function

**DOI:** 10.3389/fmicb.2024.1496280

**Published:** 2025-01-22

**Authors:** Bo Li, Haoyu Wang, Mengmeng Wang, Hewei Liang, Tongyuan Hu, Jinlong Yang, Shangyong Li, Xinbi You, Binbin Xia, Yue Yuan, Yuanqiang Zou, Yinglei Miao, Yang Sun

**Affiliations:** ^1^Department of Gastroenterology, The First Affiliated Hospital of Kunming Medical University, Kunming, China; ^2^Yunnan Province Clinical Research Center for Digestive Diseases, Kunming, China; ^3^BGI Research, Shenzhen, China; ^4^College of Life Sciences, University of Chinese Academy of Sciences, Beijing, China; ^5^School of Basic Medicine, Qingdao Medical College, Qingdao University, Qingdao, China; ^6^BGI Research, Wuhan, China; ^7^Shenzhen Engineering Laboratory of Detection and Intervention of Human Intestinal Microbiome, BGI Research, Shenzhen, China; ^8^BGI Research, Kunming, China; ^9^College of Forensic Science, Xi’an Jiaotong University, Xi’an, China; ^10^Yunnan Geriatric Medical Center, Kunming, China

**Keywords:** *Bifidobacterium adolescentis*, inflammatory bowel disease, probiotic function, intestinal barrier function, genomic analysis

## Abstract

Numerous studies have confirmed that gut microbiota is a key driver in the occurrence and progression of inflammatory bowel disease (IBD). Based on the bacterial collection constructed in our previous studies, we founded that *Bifidobacterium adolescentis* AF91-08b2A has the potential beneficial function. We designed cohort studies, genomic studies and animal experiments to further explore the probiotic function of *Bifidobacterium adolescentis* AF91-08b2A and its therapeutic effect on IBD. The depletion of *B. adolescentis* in individuals with IBD suggested its significance for intestinal health. Genomic analysis highlighted the probiotic attributes of *B. adolescentis* AF91-08b2A, including resistance to antibiotics and stress, and metabolic pathways related to energy and carbohydrate metabolism, which are likely to enhance its therapeutic efficacy. In DSS-induced mice colitis model, the strain significantly enhanced the disease activity index (DAI), curbed weight loss, and attenuated colonic damage. It effectively modulated the immune response by reducing the levels of pro-inflammatory cytokines such as IL-6, IL-1β, IL-17A, IFN-γ, and TNF-α, while promoting the secretion of anti-inflammatory cytokines like IL-4, IL-10, and TGF-β1. The restoration of tight junction proteins ZO-1, occludin, and claudin-2 by *B. adolescentis* AF91-08b2A demonstrated its capacity to safeguard the intestinal epithelial barrier. Collectively, our findings indicate *B. adolescentis* AF91-08b2A as a valuable therapeutic option for UC, with its multifaceted approach to reducing inflammation and fortifying the intestinal barrier.

## Introduction

Inflammatory bowel disease (IBD) is a chronic immune-mediated disease that primarily affects the gastrointestinal tract, including Crohn’s disease (CD) and ulcerative colitis (UC) (Chen et al., 2023). The pathological changes include inflammation of the epithelial cells in the colonic mucosa, increased mucus production, and infiltration of lymphocytes and plasma cells in the submucosa (Saez et al., 2023). These pathological changes can lead to damage to the intestinal mucosa and disturbances in function, manifesting as symptoms such as diarrhea, abdominal pain, and rectal bleeding (Le Berre et al., 2023). Long-term use of traditional drug treatment can cause high costs, the potential for drug resistance and side effects, including liver damage, osteoporosis and compromised immune system (Feng et al., 2022). Due to some shortcomings of drug therapy, some new treatment methods have been applied in recent years.

The pathogenesis of IBD is complex and involves the interaction of many factors, including genetics, immune response, environmental conditions and the imbalance of intestinal microbial balance (Caparrós et al., 2021; Nakase et al., 2022). Numerous studies indicated that IBD has been strongly associated with gut dysbiosis (Kaur et al., 2011; Sartor and Mazmanian, 2012), which involves an imbalance of beneficial microorganisms and an overgrowth of pathogenic bacteria in the intestinal microenvironment. Thus, probiotic interventions have garnered significant interest as potential therapeutic options for alleviating IBD. Research indicates that in UC patients, there is a significant shift in the composition of the gut microbiota, with a decrease in beneficial bacteria like *Bifidobacteria* and *Lactobacilli*, and an increase in potentially pathogenic species such as *E. coli* (Pittayanon et al., 2020). The metabolites from microbial sources, which are crucial for intestinal health, may become disrupted, impacting the intestinal barrier and inflammation. The immune-modulating effects of the gut microbiota are also significant, with certain probiotics capable of alleviating inflammation (Lin et al., 2024). The exploration of treatment strategies based on the gut microbiota, including probiotics, prebiotics, and fecal microbiota transplantation, is ongoing to improve UC conditions. Understanding this relationship is vital for developing new therapeutic approaches to enhance the quality of life for UC patients.

Metagenome-wide association studies (MWAS) using human stool samples, as well as animal models, have pointed to a potential role of the gut microbiome in inflammatory bowel disease (IBD) (Franzosa et al., 2018; Lewis et al., 2015; Jiang et al., 2020; He et al., 2017). *B. adolescentis* have differential abundance in IBD patients and normal people (Fan et al., 2021; Kowalska-Duplaga et al., 2019).

In previous studies, new treatments such as probiotics and prebiotics have been shown to effectively mitigate the occurrence of IBD with fewer side effects than conventional treatments (Kennedy et al., 2023; Zhao et al., 2020). With the development of clinical and animal experimental studies, *Bifidobacterium* has been shown to relieve intestinal inflammation by repairing the intestinal barrier, changing the gut microbiota, and altering cytokine levels (Fan et al., 2021; Jang et al., 2019). Intestinal mucosal damage repair is a key link to promote ulcer healing, which can radically reduce recurrence in patients and thus treat UC (Zhao et al., 2020; Ivanov and Naydenov, 2013). Tight junction (TJ) is the most important component of the mechanical barrier of intestinal mucosa, which is widely distributed between neighboring intestinal epithelial cells. Research showed that the expression of the key proteins (claudin, occludin and ZO-1) play an important role in the repair of intestinal mucosal injury (Le Berre et al., 2023). A large number of lymphocytes, neutrophils, plasma cells and macrophages gather in the colon mucosa of UC patients. Among the cytokines released by these immune cells, the imbalance between pro-inflammatory factors and anti-inflammatory factors is one of the mechanisms triggering UC (Nakase et al., 2022). A study revealed that *B. adolescentis* ameliorates colitis by reducing pro-inflammatory cytokines, modulating Treg/Th2 response, and reshaping gut microbiota composition (Fan et al., 2021).

In this study, we analyzed the abundance of cultured *B. adolescentis* in IBD patients and the health from Chinese gut metagenomic cohorts and HMP, and checked the correlation between *B. adolescentis* and other bacteria. We investigated the beneficial effects of *B. adolescentis* AF91-08b2A on intestinal barrier damage in colitis mice by measuring inflammation and tight junction protein levels. Our research indicates that *B. adolescentis* offers a protective effect on the intestinal epithelium and, furthermore, diminishes the levels of inflammatory factors.

## Materials and methods

### Metagenomic cohort collection and analysis

Human gut metagenome sequencing data of a Chinese cohort (CN_Health, a part of 4D-SZ) (Jie et al., 2021) was downloaded from the CNGB Sequence Archive (CNSA) (Guo et al., 2020)^1^ of China National GeneBank DataBase (CNGBdb) (Chen et al., 2020) under the accession code CNP0000426. Gut metagenome data of IBD patients and non-IBD controls from Guangzhou cohort was retrieved from the National Center for Biotechnology Information (NCBI) with study accession number PRJEB15371 (CN_GZ) (He et al., 2017). Gut metagenome data of IBD patients and non-IBD controls from Yunnan cohort was collected in CNSA with accession number CNP0004022 (CN_YN). Gut metagenome data of IBD patients and healthy individuals from HMP was downloaded following the link https://portal.hmpdacc.org/.

The species-level clusters of CGR2 (Lin X. et al., 2023), including *B. adolescentis*, were built as a custom genome database by Kraken v2.1.2 (Wood et al., 2019) and Bracken v2.5 (Lu et al., 2016) Kraken2 and Bracken were also used to calculate the read numbers of the species-level clusters in the cohorts established in Guangzhou, in Yunnan, and in the HMP. Relative abundances of the *B. adolescentis* in the four cohorts were calculated, and the correlations between *B. adolescentis* and other intestinal bacteria in CN_GZ, CN_YN, and HMP were analyzed based on Spearman’s rank correlation coefficient. Mean relative abundance was calculated and Wilcoxon test was used to evaluate the differential abundance of species in IBD patients and controls. Prevalence was determined using a 0.01% relative abundance threshold to indicate the presence of a cluster within a sample (Beresford-Jones et al., 2022).

### Bacterial isolation and cultivation

Strain AF91-08b2A was isolated from a fresh fecal sample obtained from a healthy female in China. Detailed methods regarding isolation and cultivation were studied by Zou et al. (2019).

### Whole-genome sequencing, assembly, and annotation

DNA extraction, sequencing, and assembly, as our previous study described (Zou et al., 2019). The genomic taxonomy of strain AF91-08b2A was determined using version 2.1.0 of GTDB-Tk, referencing with the GTDB Release 214. The genome quality of this strain was assessed by CheckM2. The Prokka was used to annotate the basic genomic information of this strain. And the genome circle map was visualized with the online Proksee server.^2^ The metabolic pathways of strain AF91-08b2A were annotated by eggNOG-mapper version 2.1.4. The carbohydrate-active enzymes (CAZymes) of strain AF91-08b2A were predicted based on the carbohydrate enzymes database using the Diamond software (--id 60 --query-cover 50 -e 0.00001). Moreover, the RGI tool with the CARD database and the virulence factor database (VFDB) with a minimum identity threshold of 60% and a query coverage exceeding 50% were used to predict the antibiotic resistance genes and virulence factors, respectively.

### Animal experiments

All experiments involving animals were conducted according to the Ethics Committee of Kunming Medical University (kmmu20241596). Six-week-old C57BL/6J mice, sourced from Guangdong Model Biotechnology Co., Ltd., were housed in a standard SPF environment. Following a one-week acclimation period, the mice were randomly assigned into four experimental groups, each comprising eight individuals: (1) a control group on a regular diet for 2 weeks; (2) a DSS group with DSS-induced colitis during the final week; (3) a 5-ASA group receiving both 5-ASA and DSS; and (4) an AF-91 group with AF-91 administration alongside DSS-induced colitis. Mice in the DSS, 5-ASA, and AF-91 groups received a 2.5% DSS solution for a period of 7 days. Administration of 5-ASA (100 mg/kg/d) and AF-91 (1 × 10^9^ CFU/d) to the mice was performed via oral gavage (Fan et al., 2021; Huang et al., 2023; Huang et al., 2022). Daily assessments of the disease activity index (DAI) were conducted for all mice. Colonic tissues were harvested on day 15, measured for length, and sectioned into three parts: one for H&E staining and immunohistochemical analysis, the remaining for molecular analysis including RT-PCR and western blotting, with storage at −80°C.

### Immunohistochemistry

Paraffin embedding and fixation of intestinal mucosal samples were performed using a 4% paraformaldehyde solution, followed by continuous sectioning. Sections were deparaffinized and rehydrated in xylene, subjected to antigen retrieval with a 0.01 M citrate buffer via microwave treatment, followed by a 10-min treatment with 3% hydrogen peroxide for peroxidase quenching at ambient temperature. Afterward, sections were incubated with primary antibodies specific for ZO-1, occludin, or claudin-2 overnight at 4°C. The specific antibodies used included anti-ZO-1 from Proteintech, anti-occludin, and anti-claudin-2, both sourced from Abcam. Subsequently, 50 μL of DAB substrate was applied to the sections for colorimetric development, with the reaction closely observed microscopically. The slides were washed twice with double steam water for 1 min each, restained with hematoxylin for 1 min, rinsed with 1% ammonia, dehydrated in 95 and 100% ethanol, sealed with neutral resin, and observed under a microscope for results.

### Histological staining

Isolated tissue specimens were immersed in a 4% paraformaldehyde solution for fixation, a process that lasted 24 h. Following this, the tissues were embedded in paraffin and subsequently sectioned. Sections were then stained using hematoxylin and eosin (H&E) to facilitate histopathological analysis.

### Reverse transcription polymerase chain reaction

The experimental procedure followed the protocol outlined in the Platinum^®^ SYBR^®^ Green qPCR SuperMix (TaKaRa, Japan). Gene expression data were normalized relative to β-actin, serving as an endogenous control. The utilized primers in this research were custom designed and synthesized by TaKaRa Bio Inc., detailed in Table 1.

**Table 1:** Primer sequences utilized in quantitative real-time RT-PCR analysis.

	Forward (5′ → 3′)	Reverse (5′ → 3′)
ZO-1	5′-GTTGGAGCCAACTCTGTTTCTCTC-3′	5′-GTTCAATCCACCTTCACATTGCTTA-3′
Occludin	5′-AAGGTCCTGGTGTGAGCTGTGA-3′	5′-AGCGCTGCTGCAAAGATTGATTAG-3′
Claudin-2	5′-ATCGGACTCAGCTGGCTTTG-3′	5′-ATCGGACTCAGCTGGCTTTG-3′
IL-6	5′-GTCGGAGGCTTAATTACACATGTTC-3′	5′-GCAAGTGCATCATCGTTGTTCA-3′
IL-10	5′-TAGAGCTGCGGACTGCCTTC-3′	5′-TGATTTCTCGCCATCCTTC-3′
TNF-α	5′-GCCTCTTCTCATTCCTGCTTGTG-3′	5′-TGATGAGAGGGAGGCCATTTG-3′
IL-1β	5′-CCAGGATGAGGACATGAGCAC-3′	5′-TGTTGTTCATCTCGGAGCCTCTA-3′
β-actin	5′-CACCATTGGCAATGAGCGGTTC-3′	5′-AGGTCTTTGCGGATGTCCACGT-3′

### Western blot analysis

Tissue specimens from mice were processed into a uniform homogenate using a lysis solution fortified with protease inhibitors at a concentration of 1%. The quantification of protein was accomplished with a specific protein quantification kit. The antibodies utilized in this study included anti-ZO-1 (Proteintech), anti-occludin (Abcam), anti-claudin-2 (Abcam), and anti-GAPDH (Proteintech). The results were analyzed using ImageJ.

### Enzyme-linked immunosorbent assay analysis

After mixing with phosphate-buffered saline (PBS, 1:9 ratio), the tissue samples were processed through homogenization and subsequent centrifugation to extract the supernatant for ELISA analysis. Quantitative assessment of both pro-inflammatory and anti-inflammatory cytokines in the colonic tissue was performed using ELISA kits procured from NeoBioscience, adhering to the protocol provided by the supplier.

### Statistical analysis

Measurement data were presented as mean ± standard deviation. Comparisons among several groups were performed utilizing one-way ANOVA through GraphPad Prism software. All graphics in this study were formatted using Adobe Illustrator. Significance indicators in the illustrations are represented with the following symbols: ^*^p < 0.05, ^**^p < 0.01, ^***^p < 0.001, and ^****^p < 0.0001.

## Results

### The distribution of *Bifidobacterium adolescentis* in metagenomic cohorts

**Figure 1:**
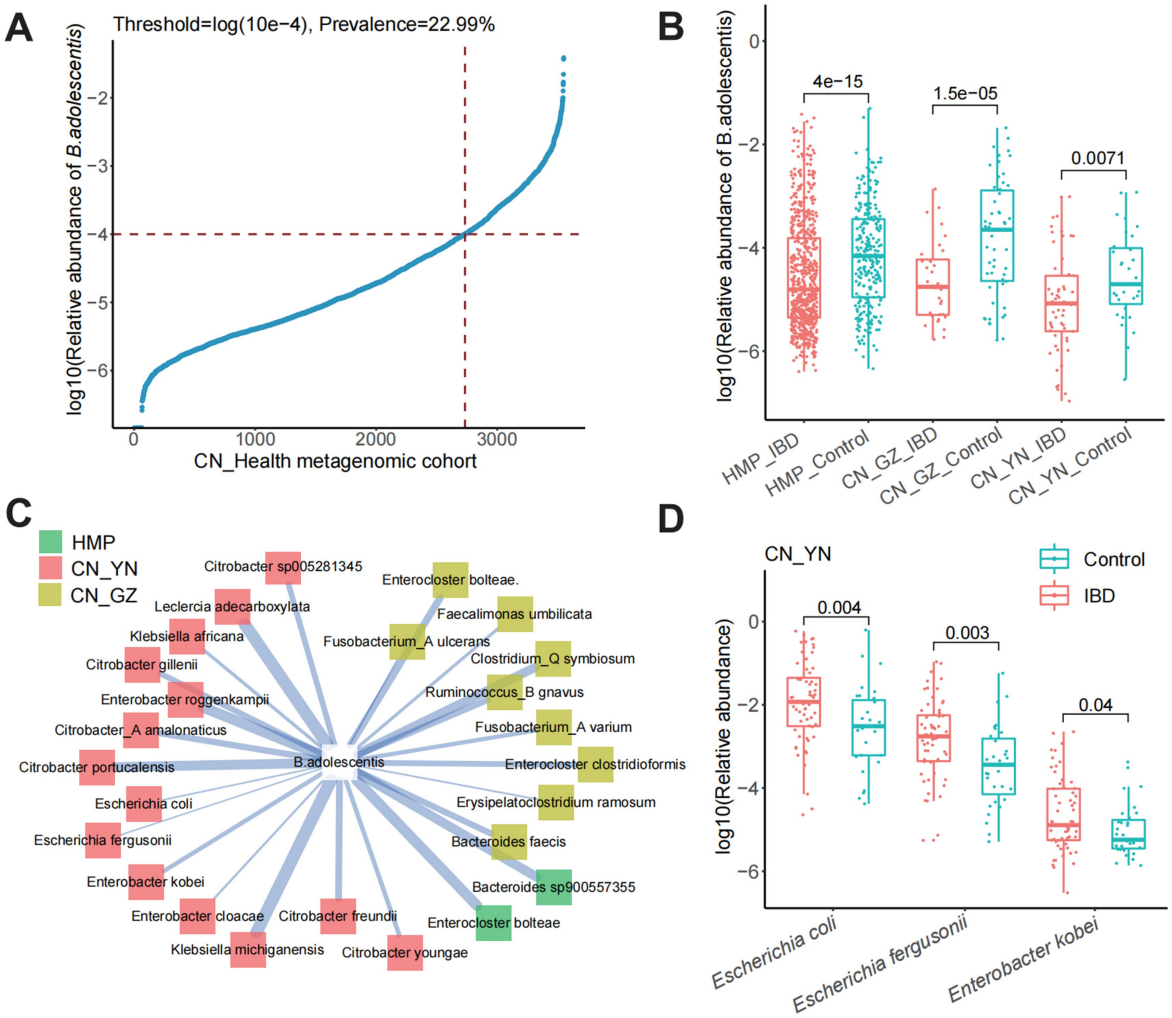
The distribution of *B. adolescentis* in metagenomic cohorts. **(A)** The occurrence rate and relative abundance of *B. adolescentis* in Chinese metagenome cohort. **(B)** Log10 relative abundance of *B. adolescentis* in IBDs and controls in metagenome cohorts of HMP, CN_GZ, and CN_YN. Mark, Wilcoxon test p-value. **(C)** Species negatively correlated with *B. adolescentis* in metagenome cohorts of CN_GZ (yellow), CN_YN (red), and HMP (green). **(D)** Log10 relative abundance of species negatively correlated with in *B. adolescentis* CN_YN. Mark, Wilcoxon test *p*-value.

*B. adolescentis* has been identified in 22.99% samples in the Chinese gut metagenomic cohort (Figure 1A). Differential abundance analysis was conducted for *B. adolescentis*. The abundance of *B. adolescentis* were lower in the intestinal of IBD patients than the health in metagenomic cohorts of HMP, CN_GZ, and CN_YN (Figure 1B). The abundance of *B. adolescentis* were lower in UC patients and in CD patients in HMP cohort (Supplementary Figure S1). The abundance of *B. adolescentis* were lower only in CD patients in CN_GZ cohort. The abundance of *B. adolescentis* were lower only in UC patients in CN_YN cohort.

To explore the potential function of *B. adolescentis* in human gut, correlation between *B. adolescentis* and intestinal bacteria was calculated. More species negatively correlated with *B. adolescentis* in CN_YN cohort, especially *Citrobacter*, including *Citrobacter freundii*, *Citrobacter gillenii*, *Citrobacter portucalensis*, *Citrobacter sp005281345*, *Citrobacter youngae*, and *Citrobacter_A amalonaticus* (Figure 1C). *Enterobacter cloacae*, *Enterobacter kobei*, and *Enterobacter roggenkampii* also negatively correlated with *B. adolescentis* in CN_YN cohort. *Enterocloster bolteae* was significantly negatively correlated with *B. adolescentis* in CN_GH cohort and HMP cohorts. In CN_GZ cohort, *Enterobacter kobei*, *Escherichia coli*, and *Escherichia fergusonii* were significantly enriched in the gut of IBD patients (Figure 1D).

### The bacterial taxonomic classification and genomic characterization

**Figure 2:**
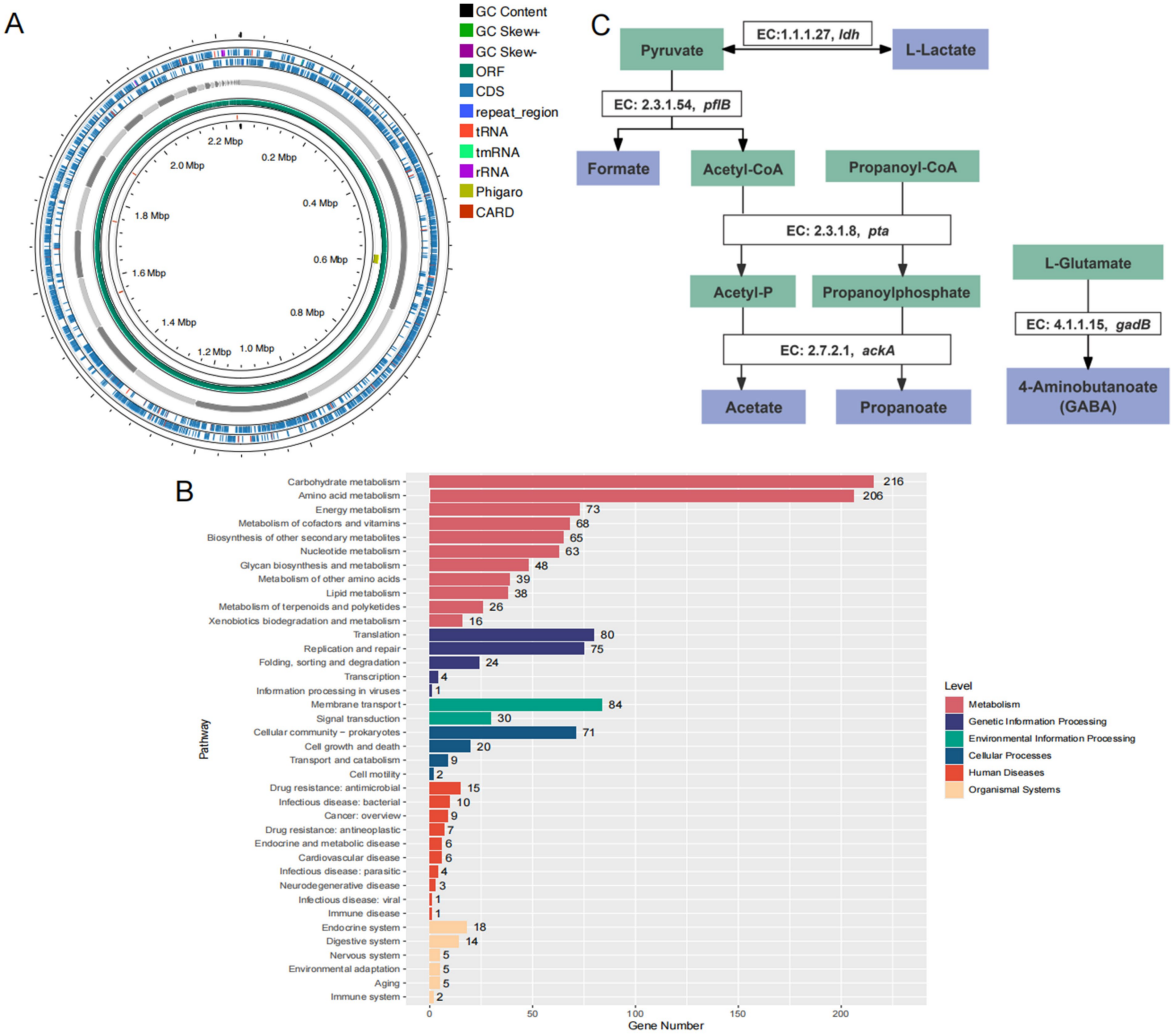
Genomic features and metabolic pathway annotations of strain AF91-08b2A. **(A)** Genomic circle map information of strain AF91-08b2A. **(B)** The KEGG metabolic pathway annotation. **(C)** Annotation of the beneficial metabolic pathways of strain AF91-08b2A.

Using the GTDB-Tk tool for whole-genome annotation, strain AF91-08b2A was classified as *B. adolescentis*, which is a species within the genus *Bifidobacterium* and the family Bifidobacteriaceae. The genome circle map of strain AF91-08b2A is shown in Figure 2A. The genome of strain AF91-08b2A has been assembled into 37 contigs, totaling 2,242,707 base pairs with a G + C content of 59.0 mol%. It was predicted to contain 1803 coding DNA sequences (CDS), 4 rRNA genes, 53 tRNA genes, and 1 tmRNA gene. The genetic function of strain AF91-08b2A was annotated by eggNOG-mapper, which showed that 1,073 genes were annotated by the Kyoto Encyclopedia of Genes and Genomes (KEGG) database, matching 911 KO and 176 pathways. Among the six categories matched by KEGG, Metabolism and Genetic Information Processing are the most abundant, which means that this strain has a strong metabolic function and environmental adaptation ability (Figure 2B). At the metabolic level, this strain has a relatively complete pathway, and multiple copies of many genes exist in some pathways, including glycolysis/gluconeogenesis, pentose phosphate pathway, galactose metabolism, starch and sucrose metabolism, amino sugar and nucleotide sugar metabolism, and other metabolic pathways. These genes were divided into six categories, and the metabolism category was the most abundant. A total of 99 genes encoding CAZymes were predicted and assigned to the four categories, glycoside transferases (GTs), carbohydrate esterases (CEs), carbohydrate-binding modules (CBMs), and glycoside hydrolases (GHs), representing 26, 4, 10, and 60%, respectively.

### Safety assessment of strain AF91-08b2A

Safety assessment of a strain typically involves a series of evaluations to ensure that the strain does not pose any health risks to humans or animals. The goal is to confirm that the strain is safe for its intended use, such as in probiotics, pharmaceuticals, or as a food additive (Di Pierro et al., 2023). In our research, we examined the genes associated with antibiotic resistance, virulence factors and toxins. As shown in the Supplementary Table S2, four antibiotic resistance genes were predicted in strain AF91-08b2A, namely *rpoB* mutants, *tet (W)*, *dfrF*, and *ErmX*. Specifically, *B. adolescentis* rpoB mutants conferring resistance to rifampicin, *tet* (*W*), *dfrF* are resistant to rifamycin antibiotic, tetracycline antibiotic, diaminopyrimidine antibiotic, respectively. *B. adolescentis rpoB* mutants conferring resistance to rifampicin and *tet (W)* are genes commonly found in *B. adolescentis*. The specific antibiotic resistance depends on the gene expression levels and the presence of sites for horizontal gene transfer. Meanwhile, a multidrug resistance gene (*ErmX*) was detected, which the gene is resistant to macrolide antibiotic, lincosamide antibiotic, streptogramin antibiotic, streptogramin A antibiotic, and streptogramin B antibiotic. Some studies have shown that the erm(*X*) gene is quite common in *Bifidobacterium* strains, and the resistance level is directly proportional to the expression of this gene. However, *Bifidobacterium breve* PRL2020 (Di Pierro et al., 2023), which contains three erm(*X*) genes and *B. adolescentis rpoB* mutants conferring resistance to rifampicin, did not demonstrate resistance to the related antibiotics *in vitro* experiments. In this strain, a total of nine virulence factor genes were predicted, which were related to bacterial adherence, immune modulation, antiphagocytosis, nutritional/metabolic factor, etc. (Supplementary Table S3). Identified as virulence factors in the VFDB due to their role in aiding the survival and colonization of pathogenic bacteria within the host, these genes, in the absence of additional pathogenic mechanisms, may be considered advantageous for probiotic function (Wu et al., 2023). Thus, none of the virulence factor gene, which is truly virulent, was identified in the genome of strain AF91-08b2A.

### Analysis of the stress-resistance genes

The annotation of the whole genome of strain AF91-08b2A was identified genes associated with probiotic properties as shown in Figure 2C. According to the whole genome sequence of AF91-08b2A, genes responsible for stress resistance include AF91-08b2A_01017 (*uspA*), AF91-08b2A_01115 (a universal stress protein family gene); genes associated with heat-shock stress resistance include AF91-08b2A_01520 (*grpE*), AF91-08b2A_01521 (*dnaK*), AF91-08b2A_00349 (*dnaJ*), AF91-08b2A_01519 (*dnaJ*), AF91-08b2A_01648, AF91-08b2A_00642 (*groS*), AF91-08b2A_01010 (*groL*), AF91-08b2A_00348 (*hrcA*); additionally, the gene AF91-08b2A_01015 (*cspB*) is responsible for cold-shock stress resistance. Furthermore, 10 genes encoding ATP synthase were related to acid resistance. They can maintain the pH stability in cells and adapt to acid environment or acid stress. Of these, studies have proved that *clpB*, *clpC*, *clpP*, and *clpX* are involved in the heat stress reaction (Wu et al., 2023; Boucard et al., 2022). Four genes were associated with anti-oxidative stress, 16 genes associated with DNA and protein protection and repair. Because of these genes, the strain AF91-08b2A can better survive in various environmental conditions.

### *Bifidobacterium adolescentis* AF91-08b2A alleviates weight loss and reduces DAI scores

**Figure 3:**
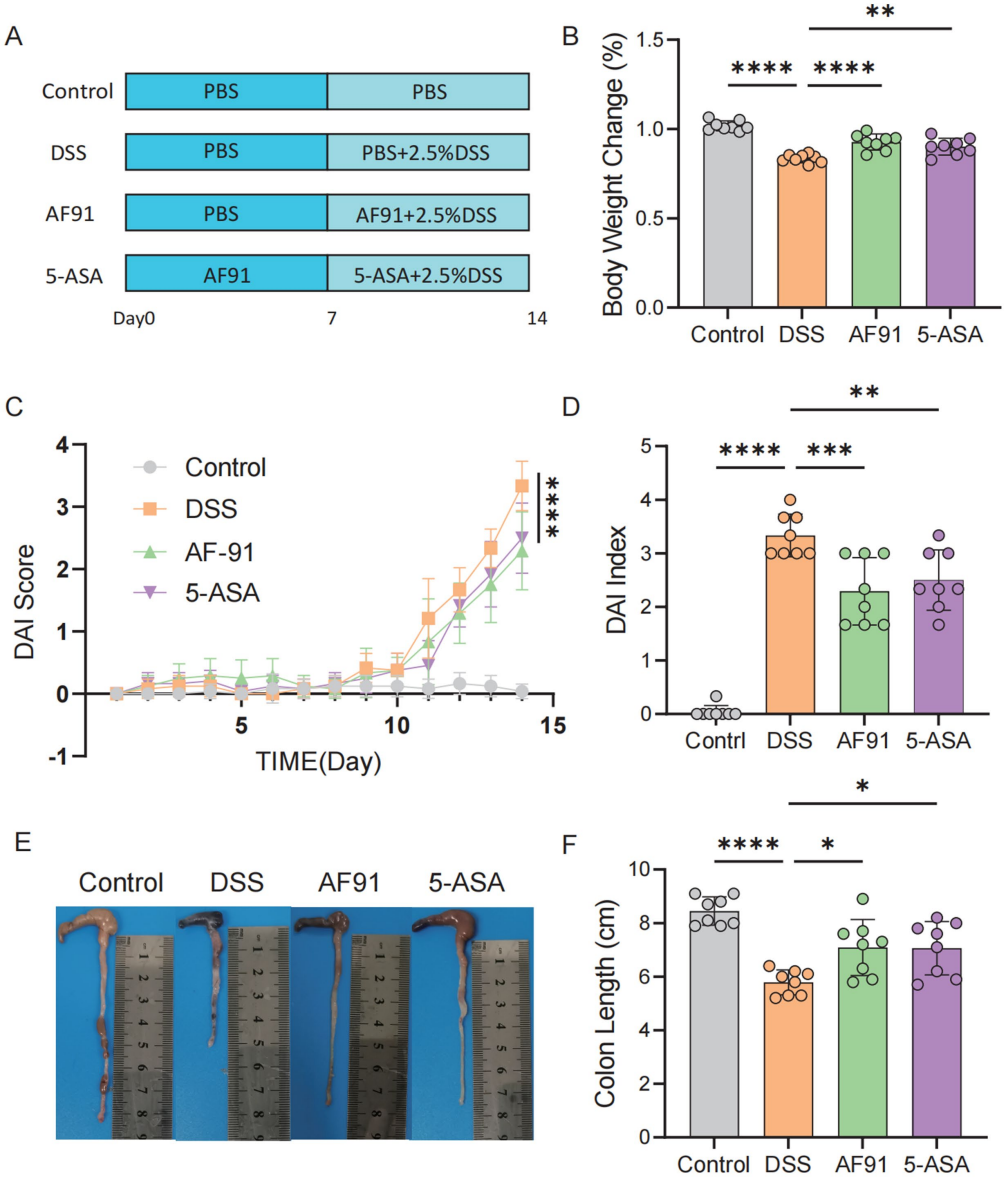
AF-91 significantly alleviated the DSS-induced clinical symptoms in mice. **(A)** The experimental design flow chart. **(B)** The changes of body weight. **(C,D)** DAI score. **(E)** Images of colon tissue. **(F)** The quantification of the colon length.

**Figure 4:**
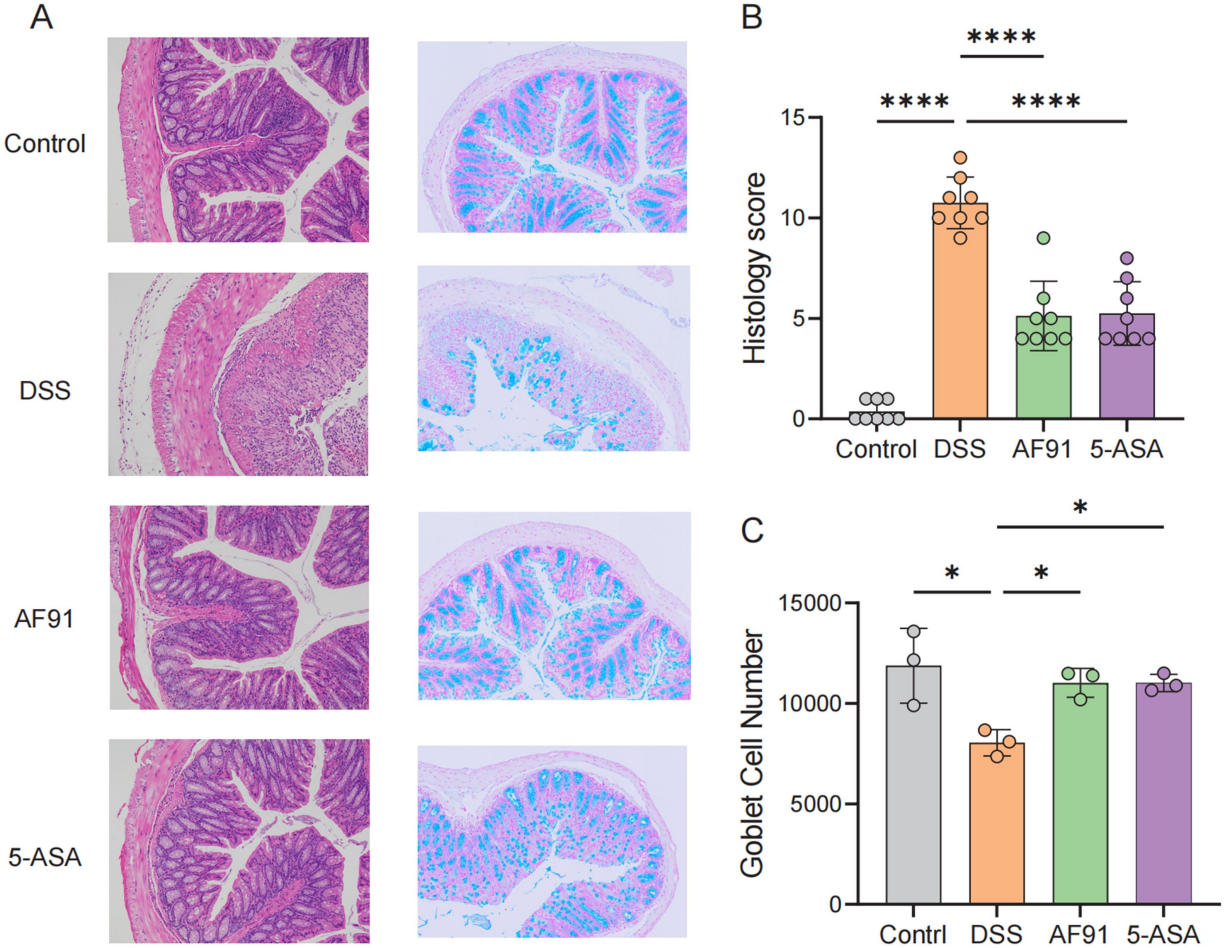
Histological analysis and goblet cell quantification in colonic tissue. **(A)** Representative micrographs of H&E staining and Alcian blue staining of colonic tissue from the four groups of mice. **(B)** Histopathology scores. **(C)** The number of goblet cells.

The animal grouping and modeling schedule were conducted as described in the Materials and Methods section (Figure 3A). To evaluate the effects of *Bifidobacterium adolescentis* AF91-08b2A on body weight changes and disease activity indices in a DSS-induced colitis mouse model, we recorded body weight alterations and disease activity indices across different treatment groups, including the control, DSS, AF91, and 5-ASA groups. The DSS group showed a significant reduction in body weight compared to the control group (*p* < 0.0001), while treatment with AF91 (*p* < 0.0001) and 5-ASA (*p* < 0.01) significantly mitigated this weight loss (Figure 3B). Moreover, the DSS group exhibited a marked increase in DAI scores from Day 10 to Day 14, reflecting severe disease activity (*p* < 0.0001). In contrast, both AF91 and 5-ASA treatments significantly reduced DAI scores compared to the DSS group, with the AF91 group showing a substantial improvement by Day 14 (*p* < 0.0001, Figures 3C,D).To further investigate the protective effects of *B. adolescentis* AF91-08b2A on colonic damage, colon lengths were measured, and histological staining was performed. The DSS group exhibited visibly shorter compared to the control group, while treatment with AF91 and 5-ASA alleviated colon shortening and preserved relatively normal morphology (Figure 3E). Quantitative measurements revealed a significant reduction in colon length in the DSS group (5.79 ± 0.46 cm) compared to the control group (8.45 ± 0.52 cm), as shown in Figure 3F (*p* < 0.0001). Notably, the AF91 group exhibited a significantly longer colon length (7.15 ± 0.86 cm) than the DSS group (*p* < 0.05, Figure 3F), suggesting that *B. adolescentis* AF91-08b2A could attenuate DSS-induced colonic shortening. Histological analysis of H&E-stained colon sections further supported these findings. In the control group, intestinal mucosal epithelial cells were intact and organized, with no evidence of necrosis, shedding, or inflammatory cell infiltration. In contrast, DSS treatment induced submucosal edema, epithelial cell porosity, crypt damage, and significant inflammatory cell infiltration (Figure 4A). Both AF91 and 5-ASA treatment groups exhibit significantly reduced scores compared to the DSS group, suggesting their therapeutic effects (*p* < 0.0001, Figure 4B).These results suggest that *B. adolescentis* AF91-08b2A could effectively mitigate DSS-induced intestinal tissue damage.

### *Bifidobacterium adolescentis* AF91-08b2A recovered goblet cells exhaustion

The content of intestinal goblet cells was analyzed by Alcian blue staining to further verify the alleviating effect of the strain on intestinal barrier destruction. Goblet cells were abundant in the control group (Figure 4A), mainly distributed on the surface of colon epithelial cells. In contrast, in the DSS group, the number of goblet cells was significantly reduced due to damage to the inner and outer mucosal layers of the colon, however, goblet cell counts were significantly improved with 5-ASA and AF91 groups (*p* < 0.05, Figure 4C).

### AF91 upregulates anti-inflammatory cytokines and downregulates pro-inflammatory cytokines in DSS-induced colitis mice

**Figure 5:**
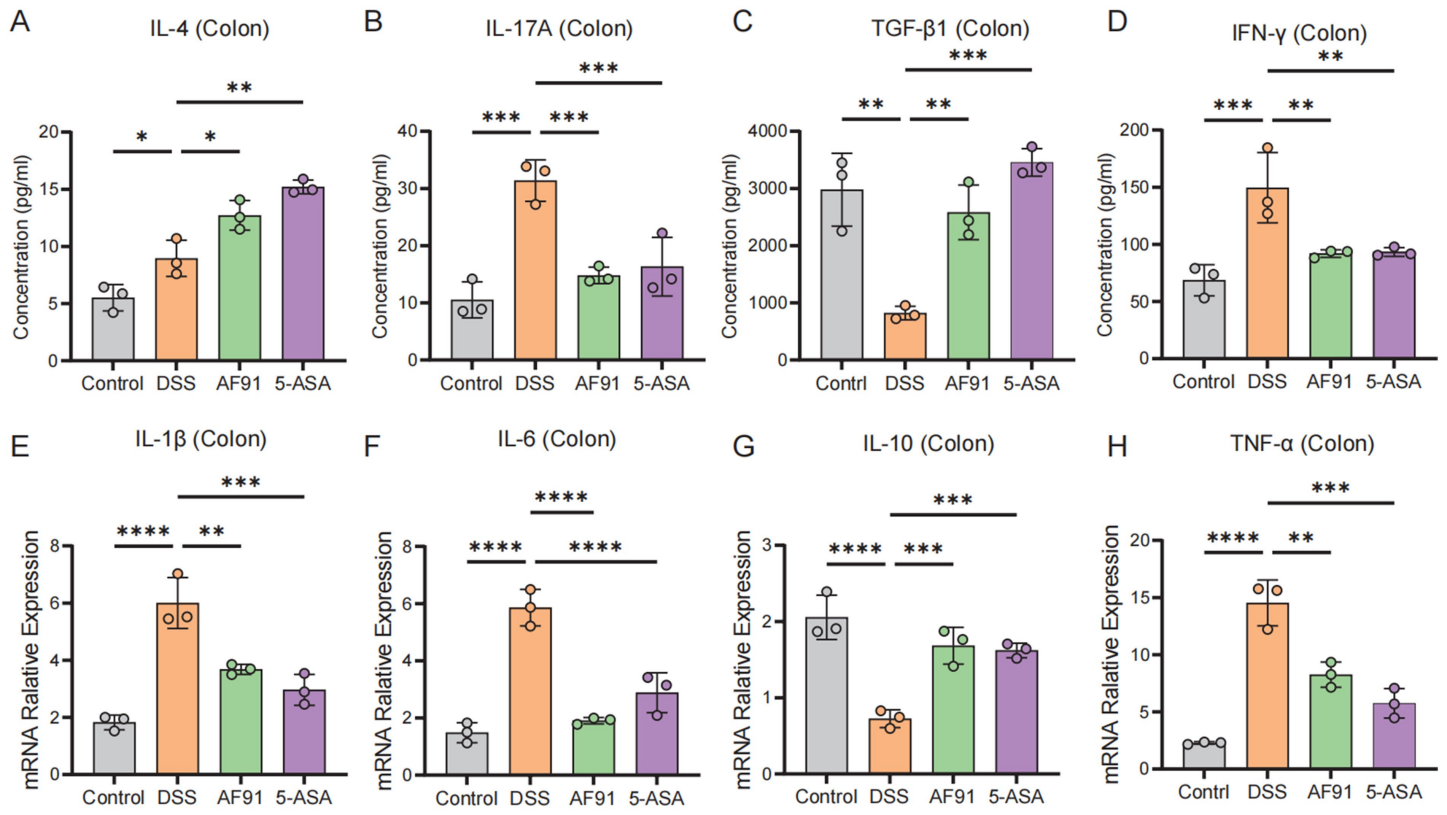
Effects of AF-91 on pro-inflammatory and anti-inflammatory cytokine expression. **(A–D)** The levels of IL-4 **(A)**, IL-17a **(B)**, TGF-β1 **(C)** and IFN-γ **(D)** in colon tissues were determined using ELISA. **(E–H)** The relative mRNA expression levels of IL-1β **(E)**, IL-6 **(F)**, IL-10 **(G)**, TNF-α **(H)**.

Colonic cytokine levels are indicative of the extent of inflammation triggered by DSS. Assessment of colonic damage included quantifying levels of the proinflammatory cytokines IFN-γ and IL-17A, and the anti-inflammatory cytokines IL-4 and TGF-β1. Our data indicate a significant downregulation of IL-4 and TGF-β1, and upregulation of IFN-γ and IL-17A in the DSS group versus the control (Figures 5A,C vs. Figures 5B,D). Elevated levels of IL-6, IL-1β, and TNF-α, along with reduced IL-10, were observed in the DSS group’s colonic tissue compared to the control, with statistical significance (*p* < 0.05) (Figures 5E–H). Administration of 5-ASA and AF91 led to notable amelioration of these cytokine levels. Conversely, the AF91 and 5-ASA groups demonstrated an opposing cytokine profile. These findings indicate that AF91 mitigated DSS-induced intestinal inflammation by reducing pro-inflammatory cytokines and enhancing anti-inflammatory cytokine expression.

### AF91 improves the destruction of the intestinal barrier induced by DSS

**Figure 6:**
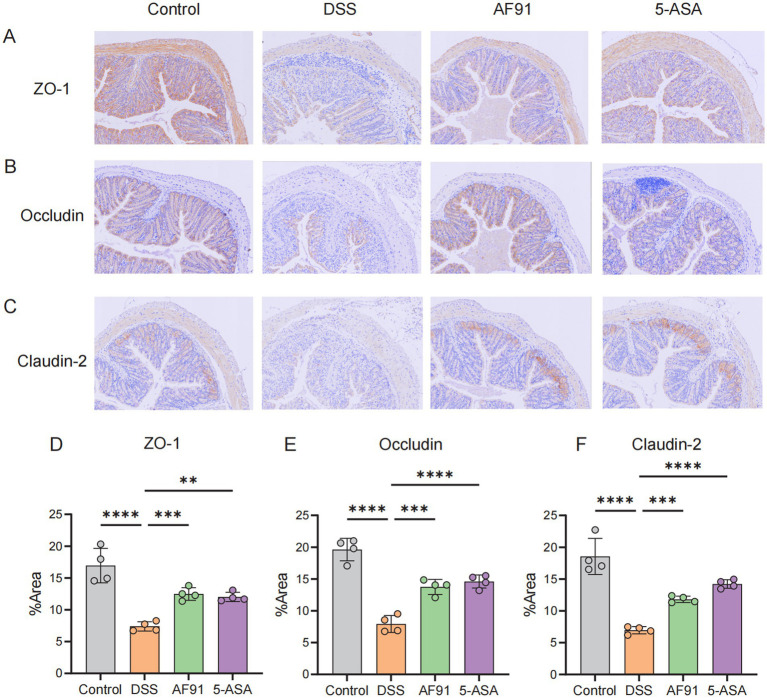
Effects of AF-91 on the epithelial barrier. The IHC staining of TJ protein **(A–C)** and area % of TJ protein **(D–F)**.

**Figure 7:**
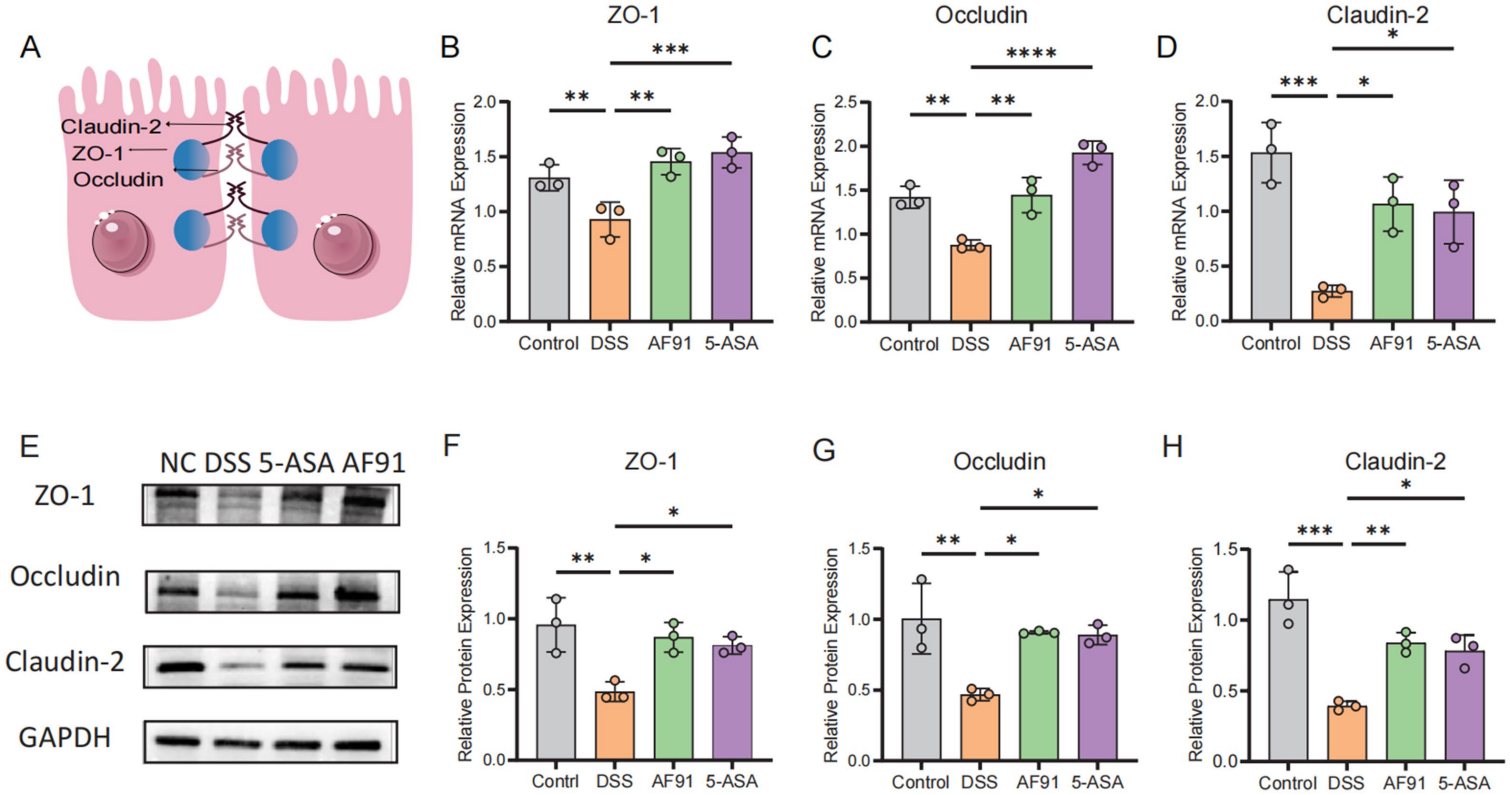
Effects of AF-91 on the intestinal TJs. Schematic representation of the intestinal tight junctions. **(A)** The relative mRNA expression levels of ZO-1 **(B)**, occludin **(C)**, and claudin-2 **(D)**. The relative protein expression levels of ZO-1 **(F)**, occludin **(G)** and claudin-2 **(H)**. Western blot analysis of ZO-1, occludin and claudin-2 **(E)**.

To further explore how AF91 modulates the expression of proteins critical to the intestinal barrier, we employed immunohistochemistry, western blot analysis, and RT-qPCR to assess the expression of intestinal barrier proteins (Figures 6, 7). DSS treatment diminished the mRNA levels of these barrier proteins (Figures 7B–D), with AF91 partially recuperating these levels. Furthermore, protein levels of ZO-1, occludin, and claudin-2 were markedly decreased in the DSS-exposed group as opposed to the control cohort, whereas AF91 supplementation partially reversed the DSS-induced reduction in expression (Figures 7F–H). Our findings indicate that AF91 may mitigate inflammation by preserving the structural integrity of the intestinal barrier.

## Discussion

These are accumulating evidences showed that gut microbiota has closely correlation to human health, especially as a key participant of inflammatory bowel disease (IBD) and other gastrointestinal diseases (Lavelle and Sokol, 2020; Wong and Yu, 2023; Stewart et al., 1977). *B. adolescentis*, one of the most abundant *Bifidobacterium* in human colon, which is common in 60–80% of healthy adults and usually applied in the treatment of constipation, irritable bowel syndrome (IBS) and IBD (Gavzy et al., 2023; He et al., 2023). Moreover, *B. adolescentis* was proved the anti-virus ability potential caused by its Mx GTPase pathway (Lee et al., 2013). In previous study, we isolated a *B. adolescentis* strain named AF91-08b2A (Lin X. et al., 2023). The whole genome sequencing was used to build the genome landscape of *B. adolescentis* AF91-08b2A, while we found that *B. adolescentis* has a wide range of sugar transporters and degrading enzyme libraries (Begley et al., 2005; Wei et al., 2023; Sonnenburg et al., 2005). Additionally, we noticed that many metabolic pathways of *B. adolescentis* AF91-08b2A including carbohydrate metabolism, amino acid metabolism, vitamin metabolism were enriched, pointing this strain has the potential of fermenting plant derived glycans and producing beneficial metabolites like most *B. adolescentis* strains (Leser and Baker, 2023). Recently reports also revealed that *B. adolescentis* has the ability of alleviating colitis and improving immune microenvironment (Fan et al., 2021; Jang et al., 2019; Lin Y. et al., 2023).

To further explore the role of *B. adolescentis* in IBD, we collected metagenomic sequencing data of three cohort studies from HMP2, CN_GZ and CN_YN. These results showed that *B. adolescentis* decreased in IBD groups compared with control groups and other reports also suggested this change of *B. adolescentis* (Fan et al., 2021; Su et al., 2023).

Considering the significant relative abundance differences in cohort studies and advantages in the genome of *B. adolescentis*, we performed comprehensive experiments to study the effect of *B. adolescentis* AF91-08b2A in DSS-induced colitis. Here, we observed that treatment of *B. adolescentis* AF91-08b2A prevented DSS-induced colitis and the decrease of body weight in mice. Gavage with *B. adolescentis* AF91-08b2A further suppressed local and systemic inflammation, especially increasing the expression level of TGF-β1 and decreasing the expression level of IL-17A, which was considered maintaining the balance of Treg/Th17 cells (Wang et al., 2022; Wen et al., 2021). IL-4 was reported as a well-known anti-inflammation factor producing by Th2 cells and our result showed the increase of IL-4 (Bosurgi et al., 2017).

The damage of intestinal epithelial barrier will increase the chance of pathogen invasion, thus accelerating the occurrence and development of IBD (Chen et al., 2021; Niu et al., 2022). Mucus layer was considered protecting intestinal immune system from excessive bacterial invasion and a key part of intestinal epithelial barrier (Kudelka et al., 2020). Supplementation of *B. adolescentis* AF91-08b2A improved gut barrier function and secretion of mucin proteins. As an important contributor of intestinal epithelial barrier, the dysfunction of tight junction (TJ) proteins aggravates the inflammatory reaction (Suzuki, 2020). Therefore, we detected three TJ proteins (ZO-1, occludin, claudin-2) and *B. adolescentis* AF91-08b2A reversed the decrease of TJ expression induced by DSS. The above results suggested that *B. adolescentis* AF91-08b2A helped intestinal epithelial healing and maintained the normal function of intestinal epithelial barrier.

Several metabolites produced by the gut microbiota, including short chain fatty acids (SCFAs), are crucial for host metabolism, energy balance, and immune system development (Flint et al., 2015; Mann et al., 2024; Martin-Gallausiaux et al., 2021). SCFAs also serve as activators of receptors on host epithelial cells and liver cells, functioning as histone deacetylase inhibitors that regulate gene expression and physiological responses in the host (Martin-Gallausiaux et al., 2021). Among SCFAs, acetate and lactate are major members for modulating intestinal epithelial cells (IECs) (Ardawi and Newsholme, 1985; Fleming et al., 1991) and can help maintain the homeostasis of the intestinal environment. Previous studies have proved that *B. adolescentis* produced a large amount of acetate and lactate through *in vitro* fermentation (Leser and Baker, 2023). Therefore, it is plausible that some of the effects of administering *B. adolescentis* AF91-08b2A are dependent on acetate and lactate production in the intestines.

The analysis of the cohorts, genome mining of function and mice experiment verification was used to systematically explore the probiotic function of *B. adolescentis* AF91-08b2A, providing an important basis for the subsequent application of this strain in products. Nevertheless, we still lack the exploration of the mechanism by which *B. adolescentis* AF91-08b2A alleviates colitis in mice. The relative abundance of *B. adolescentis* AF91-08b2A is not clear, hindering us from knowing whether colonization. In subsequent studies, we will use metabolomics and single-cell transcriptomics to further explore the mechanism of *B. adolescentis* AF91-08b2A treating colitis. Our study provides a safe and effective approach for the prevention and treatment of colitis.

## Conclusion

In conclusion, our study revealed the potential probiotic properties of *B. adolescentis* AF91-08b2A through whole genome and cohort analysis. These results also point to an important role of *B. adolescentis* AF91-08b2A in preventing DSS-induced colitis and local and systemic inflammation, indicating that *B. adolescentis* AF91-08b2A has the potential for being a probiotic supplement.

## Data Availability

The data presented in the study are deposited in the China National GeneBank DataBase, (CNGBdb), accession number is CNP0000426.

## Footnotes

^1^ https://db.cngb.org/cnsa/

^2^ https://proksee.ca/

## References

[ref1] Ardawi M. S. M. Newsholme E. A. (1985). Fuel utilization in colonocytes of the rat. Biochem. J. 231, 713–719. doi: 10.1042/bj2310713, PMID: 4074334 PMC1152807

[ref2] Begley M. Gahan C. G. M. Hill C. (2005). The interaction between bacteria and bile. FEMS Microbiol. Rev. 29, 625–651. doi: 10.1016/j.femsre.2004.09.003, PMID: 16102595

[ref3] Beresford-Jones B. S. Forster S. C. Stares M. D. Notley G. Viciani E. Browne H. P.. (2022). The mouse gastrointestinal bacteria catalogue enables translation between the mouse and human gut microbiotas via functional mapping. Cell Host Microbe 30, 124–138.e8. doi: 10.1016/j.chom.2021.12.003, PMID: 34971560 PMC8763404

[ref4] Bosurgi L. Grace Cao Y. Cabeza-Cabrerizo M. Tucci A. Hughes L. D. Kong Y.. (2017). Macrophage function in tissue repair and remodeling requires IL-4 or IL-13 with apoptotic cells. Science 356, 1072–1076. doi: 10.1126/science.aai8132, PMID: 28495875 PMC5556699

[ref5] Boucard A.-S. Florent I. Polack B. Langella P. Bermúdez-Humarán L. G. (2022). Genome sequence and assessment of safety and potential probiotic traits of *Lactobacillus johnsonii* CNCM I-4884. Microorganisms 10:273. doi: 10.3390/microorganisms10020273, PMID: 35208728 PMC8876136

[ref6] Caparrós E. Wiest R. Scharl M. Rogler G. Casbas A. G. Yilmaz B.. (2021). Dysbiotic microbiota interactions in Crohn’s disease. Gut Microbes 13:1949096. doi: 10.1080/19490976.2021.1949096, PMID: 34313550 PMC8320851

[ref7] Chen Y. Cui W. Li X. Yang H. (2021). Interaction between commensal bacteria, immune response and the intestinal barrier in inflammatory bowel disease. Front. Immunol. 12:761981. doi: 10.3389/fimmu.2021.761981, PMID: 34858414 PMC8632219

[ref8] Chen M. Yao H. Tan H. Huang W. Quanyong W. Nie S. (2023). Impact of *Bifidobacterium longum* NSP001 on DSS-induced colitis in conventional and humanised mice. Food Sci. Human Wellness 12, 1109–1118. doi: 10.1016/j.fshw.2022.10.028

[ref9] Chen F. Z. You L. J. Yang F. Wang L. N. Guo X. Q. Gao F.. (2020). CNGBdb: China National GeneBank DataBase. Yi Chuan 42, 799–809. doi: 10.16288/j.yczz.20-080, PMID: 32952115

[ref10] Di Pierro F. Campedelli I. De Marta P. Fracchetti F. Del Casale A. Cavecchia I.. (2023). *Bifidobacterium breve* PRL2020: antibiotic-resistant profile and genomic detection of antibiotic resistance determinants. Microorganisms 11:1649. doi: 10.3390/microorganisms11071649, PMID: 37512822 PMC10383950

[ref11] Fan L. Qi Y. Siwen Q. Chen X. Li A. Hendi M.. (2021). *B. adolescentis* ameliorates chronic colitis by regulating Treg/Th2 response and gut microbiota remodeling. Gut Microbes 13, 1–17. doi: 10.1080/19490976.2020.1826746, PMID: 33557671 PMC7889144

[ref12] Feng C. Zhang W. Zhang T. He Q. Kwok L.-Y. Tan Y.. (2022). Heat-killed *Bifidobacterium bifidum* B1628 may alleviate dextran sulfate sodium-induced colitis in mice, and the anti-inflammatory effect is associated with gut microbiota modulation. Nutrients 14:5233. doi: 10.3390/nu14245233, PMID: 36558391 PMC9784753

[ref13] Fleming S. E. Choi S. Y. Fitch M. D. (1991). Absorption of short-chain fatty acids from the rat cecum *in vivo*. J. Nutr. 121, 1787–1797. doi: 10.1093/jn/121.11.1787, PMID: 1941187

[ref14] Flint H. J. Duncan S. H. Scott K. P. Louis P. (2015). Links between diet, gut microbiota composition and gut metabolism. Proc. Nutr. Soc. 74, 13–22. doi: 10.1017/S0029665114001463, PMID: 25268552

[ref15] Franzosa E. A. Sirota-Madi A. Avila-Pacheco J. Fornelos N. Haiser H. J. Reinker S.. (2018). Gut microbiome structure and metabolic activity in inflammatory bowel disease. Nat. Microbiol. 4, 293–305. doi: 10.1038/s41564-018-0306-4, PMID: 30531976 PMC6342642

[ref16] Gavzy S. J. Kensiski A. Lee Z. L. Mongodin E. F. Ma B. Bromberg J. S. (2023). Bifidobacterium mechanisms of immune modulation and tolerance. Gut Microbes 15:2291164. doi: 10.1080/19490976.2023.2291164, PMID: 38055306 PMC10730214

[ref17] Guo X. Chen F. Gao F. Li L. Liu K. You L.. (2020). CNSA: a data repository for archiving omics data. Database 2020:baaa055. doi: 10.1093/database/baaa055 32705130 PMC7377928

[ref18] He Q. Gao Y. Jie Z. Xinlei Y. Laursen J. M. Xiao L.. (2017). Two distinct metacommunities characterize the gut microbiota in Crohn’s disease patients. GigaScience 6:gix050. doi: 10.1093/gigascience/gix050, PMID: 28655159 PMC5624284

[ref19] He B.-L. Xiong Y. Teng-Gen H. Zong M.-H. Hong W. (2023). *Bifidobacterium* spp. as functional foods: a review of current status, challenges, and strategies. Crit. Rev. Food Sci. Nutr. 63, 8048–8065. doi: 10.1080/10408398.2022.2054934, PMID: 35319324

[ref20] Huang C. Hao W. Wang X. Zhou R. Lin Q. (2023). Probiotics for the treatment of ulcerative colitis: a review of experimental research from 2018 to 2022. Front. Microbiol. 14:1211271. doi: 10.3389/fmicb.2023.1211271, PMID: 37485519 PMC10358780

[ref21] Huang L. Zheng J. Sun G. Yang H. Sun X. Yao X.. (2022). 5-aminosalicylic acid ameliorates dextran sulfate sodium-induced colitis in mice by modulating gut microbiota and bile acid metabolism. Cell. Mol. Life Sci. 79:460. doi: 10.1007/s00018-022-04471-3, PMID: 35913641 PMC11071811

[ref22] Ivanov A. I. Naydenov N. G. (2013). Dynamics and regulation of epithelial adherens junctions: recent discoveries and controversies. Int. Rev. Cell Mol. Biol. 303, 27–99. doi: 10.1016/B978-0-12-407697-6.00002-7, PMID: 23445808

[ref23] Jang H.-M. Lee K.-E. Kim D.-H. (2019). The preventive and curative effects of *Lactobacillus reuteri* NK33 and *Bifidobacterium adolescentis* NK98 on immobilization stress-induced anxiety/depression and colitis in mice. Nutrients 11:819. doi: 10.3390/nu11040819, PMID: 30979031 PMC6521032

[ref24] Jiang P. Lai S. Sicheng W. Zhao X.-M. Chen W.-H. (2020). Host DNA contents in fecal metagenomics as a biomarker for intestinal diseases and effective treatment. BMC Genomics 21:348. doi: 10.1186/s12864-020-6749-z, PMID: 32393180 PMC7216530

[ref25] Jie Z. Liang S. Ding Q. Li F. Tang S. Wang D.. (2021). A transomic cohort as a reference point for promoting a healthy human gut microbiome. Med. Microecol. 8:100039. doi: 10.1016/j.medmic.2021.100039, PMID: 39713500

[ref26] Kaur N. Chen C.-C. Luther J. Kao J. Y. (2011). Intestinal dysbiosis in inflammatory bowel disease. Gut Microbes 2, 211–216. doi: 10.4161/gmic.2.4.17863, PMID: 21983063

[ref27] Kennedy J. M. De Silva A. Walton G. E. Gibson G. R. (2023). A review on the use of prebiotics in ulcerative colitis. Trends Microbiol. 32, 507–515. doi: 10.1016/j.tim.2023.11.007, PMID: 38065786

[ref28] Kowalska-Duplaga K. Gosiewski T. Kapusta P. Sroka-Oleksiak A. Wędrychowicz A. Pieczarkowski S.. (2019). Differences in the intestinal microbiome of healthy children and patients with newly diagnosed Crohn’s disease. Sci. Rep. 9:18880. doi: 10.1038/s41598-019-55290-9, PMID: 31827191 PMC6906406

[ref29] Kudelka M. R. Stowell S. R. Cummings R. D. Neish A. S. (2020). Intestinal epithelial glycosylation in homeostasis and gut microbiota interactions in IBD. Nat. Rev. Gastroenterol. Hepatol. 17, 597–617. doi: 10.1038/s41575-020-0331-7, PMID: 32710014 PMC8211394

[ref30] Lavelle A. Sokol H. (2020). Gut microbiota-derived metabolites as key actors in inflammatory bowel disease. Nat. Rev. Gastroenterol. Hepatol. 17, 223–237. doi: 10.1038/s41575-019-0258-z, PMID: 32076145

[ref31] Le Berre C. Honap S. Peyrin-Biroulet L. (2023). Ulcerative colitis. Lancet 402, 571–584. doi: 10.1016/S0140-6736(23)00966-2, PMID: 37573077

[ref32] Lee D. K. Kang J. Y. Shin H. S. Park I. H. Ha N. J. (2013). Antiviral activity of *Bifidobacterium adolescentis* SPM0212 against hepatitis B virus. Arch. Pharm. Res. 36, 1525–1532. doi: 10.1007/s12272-013-0141-3, PMID: 23657805

[ref33] Leser T. Baker A. (2023). *Bifidobacterium adolescentis*—a beneficial microbe. Benefic. Microbes 14, 525–551. doi: 10.1163/18762891-20230030, PMID: 38350464

[ref34] Lewis J. D. Chen E. Z. Baldassano R. N. Otley A. R. Griffiths A. M. Lee D.. (2015). Inflammation, antibiotics, and diet as environmental stressors of the gut microbiome in pediatric Crohn’s disease. Cell Host Microbe 18, 489–500. doi: 10.1016/j.chom.2015.09.008, PMID: 26468751 PMC4633303

[ref35] Lin Y. Fan L. Qi Y. Chaochao X. Jia D. Jiang Y.. (2023). *Bifidobacterium adolescentis* induces Decorin^+^ macrophages via TLR2 to suppress colorectal carcinogenesis. J. Exp. Clin. Cancer Res. 42:172. doi: 10.1186/s13046-023-02746-6, PMID: 37464382 PMC10353206

[ref36] Lin X. Tongyuan H. Chen J. Liang H. Zhou J. Zhinan W.. (2023). The genomic landscape of reference genomes of cultivated human gut bacteria. Nat. Commun. 14:1663. doi: 10.1038/s41467-023-37396-x, PMID: 36966151 PMC10039858

[ref37] Lin X. Tongyuan H. Zhinan W. Li L. Wang Y. Wen D.. (2024). Isolation of potentially novel species expands the genomic and functional diversity of Lachnospiraceae. iMeta 3:e174. doi: 10.1002/imt2.174, PMID: 38882499 PMC11170972

[ref38] Lu J. Breitwieser F. P. Thielen P. Salzberg S. L. (2016). Bracken: estimating species abundance in metagenomics data. PeerJ Comput. Sci. 3:e104. doi: 10.1101/051813 PMC12016282 40271438

[ref39] Mann E. R. Lam Y. K. Uhlig H. H. (2024). Short-chain fatty acids: linking diet, the microbiome and immunity. Nat. Rev. Immunol. 24, 577–595. doi: 10.1038/s41577-024-01014-8, PMID: 38565643

[ref40] Martin-Gallausiaux C. Marinelli L. Blottière H. M. Larraufie P. Lapaque N. (2021). SCFA: mechanisms and functional importance in the gut. Proc. Nutr. Soc. 80, 37–49. doi: 10.1017/S0029665120006916, PMID: 32238208

[ref41] Nakase H. Sato N. Mizuno N. Ikawa Y. (2022). The influence of cytokines on the complex pathology of ulcerative colitis. Autoimmun. Rev. 21:103017. doi: 10.1016/j.autrev.2021.103017, PMID: 34902606

[ref42] Niu M.-M. Guo H.-X. Cai J.-W. Meng X.-C. (2022). *Bifidobacterium breve* alleviates DSS-induced colitis in mice by maintaining the mucosal and epithelial barriers and modulating gut microbes. Nutrients 14:3671. doi: 10.3390/nu14183671, PMID: 36145047 PMC9503522

[ref9001] Pittayanon R. Lau J. T. Leontiadis G. I. Tse F. Yuan Y. Surette M.. (2020). Differences in Gut Microbiota in Patients With vs Without Inflammatory Bowel Diseases: A Systematic Review. Gastroenterology, 158:930–946.e1. doi: 10.1053/j.gastro.2019.11.294, PMID: 31812509

[ref43] Saez A. Herrero-Fernandez B. Gomez-Bris R. Sánchez-Martinez H. Gonzalez-Granado J. M. (2023). Pathophysiology of inflammatory bowel disease: innate immune system. Int. J. Mol. Sci. 24:1526. doi: 10.3390/ijms24021526, PMID: 36675038 PMC9863490

[ref44] Sartor R. B. Mazmanian S. K. (2012). The intestinal microbiota in inflammatory bowel diseases. Am. J. Gastroenterol. Suppl. 1, 15–21. doi: 10.1038/ajgsup.2012.4

[ref45] Sonnenburg J. L. Jian X. Leip D. D. Chen C.-H. Westover B. P. Weatherford J.. (2005). Glycan foraging *in vivo* by an intestine-adapted bacterial symbiont. Science 307, 1955–1959. doi: 10.1126/science.1109051, PMID: 15790854

[ref46] Stewart W. E. Lin L. Chudzio T. Wiranowska-Stewart M. (1977). Molecular alterations of interferons. Tex. Rep. Biol. Med. 35, 193–197. 358448

[ref47] Su K.-W. Cetinbas M. Martin V. M. Virkud Y. V. Seay H. Ndahayo R.. (2023). Early infancy dysbiosis in food protein-induced enterocolitis syndrome: a prospective cohort study. Allergy 78, 1595–1604. doi: 10.1111/all.15644, PMID: 36635218 PMC10534226

[ref48] Suzuki T. (2020). Regulation of the intestinal barrier by nutrients: the role of tight junctions. Anim. Sci. J. 91:e13357. doi: 10.1111/asj.13357, PMID: 32219956 PMC7187240

[ref49] Wang H. Liu N. Yang Z. Zhao K. Pang H. Shao K.. (2022). Preventive effect of pectic oligosaccharides on acute colitis model mice: modulating epithelial barrier, gut microbiota and Treg/Th17 balance. Food Funct. 13, 9999–10012. doi: 10.1039/d2fo01448c, PMID: 36065954

[ref50] Wei X. Leilei Y. Zhang C. Ni Y. Zhang H. Zhai Q.. (2023). Genetic-phenotype analysis of *Bifidobacterium bifidum* and its glycoside hydrolase gene distribution at different age groups. Foods 12:922. doi: 10.3390/foods12050922, PMID: 36900439 PMC10000437

[ref51] Wen S. He L. Zhong Z. Zhao R. Weng S. Mi H.. (2021). Stigmasterol restores the balance of Treg/Th17 cells by activating the butyrate-PPARγ axis in colitis. Front. Immunol. 12:741934. doi: 10.3389/fimmu.2021.741934, PMID: 34691046 PMC8526899

[ref52] Wong C. C. Yu J. (2023). Gut microbiota in colorectal cancer development and therapy. Nat. Rev. Clin. Oncol. 20, 429–452. doi: 10.1038/s41571-023-00766-x, PMID: 37169888

[ref53] Wood D. E. Jennifer L. Langmead B. (2019). Improved metagenomic analysis with Kraken 2. Genome Biol. 20:257. doi: 10.1186/s13059-019-1891-0, PMID: 31779668 PMC6883579

[ref54] Wu J.-J. Zhou Q.-Y. Liu D.-M. Xiong J. Liang M.-H. Tang J.. (2023). Evaluation of the safety and probiotic properties of *Lactobacillus gasseri* LGZ1029 based on whole genome analysis. LWT 184:114759. doi: 10.1016/j.lwt.2023.114759, PMID: 39713500

[ref55] Zhao Z. Chen L. Zhao Y. Wang C. Duan C. Yang G.. (2020). *Lactobacillus Plantarum* NA136 ameliorates nonalcoholic fatty liver disease by modulating gut microbiota, improving intestinal barrier integrity, and attenuating inflammation. Appl. Microbiol. Biotechnol. 104, 5273–5282. doi: 10.1007/s00253-020-10633-9, PMID: 32335723

[ref56] Zou Y. Xue W. Luo G. Deng Z. Qin P. Guo R.. (2019). 1,520 reference genomes from cultivated human gut bacteria enable functional microbiome analyses. Nat. Biotechnol. 37, 179–185. doi: 10.1038/s41587-018-0008-8, PMID: 30718868 PMC6784896

