# Supplementary material for: Genome analysis of Bifidobacterium adolescentis and investigation of its effects on inflammation and intestinal barrier function
Source: Front Microbiol. 2025 Jan 22;15:1496280. doi: 10.3389/fmicb.2024.1496280 (PMC11794259; doi:10.3389/fmicb.2024.1496280)
Supplement: Supplementary file 1 [file Data_Sheet_1.docx]

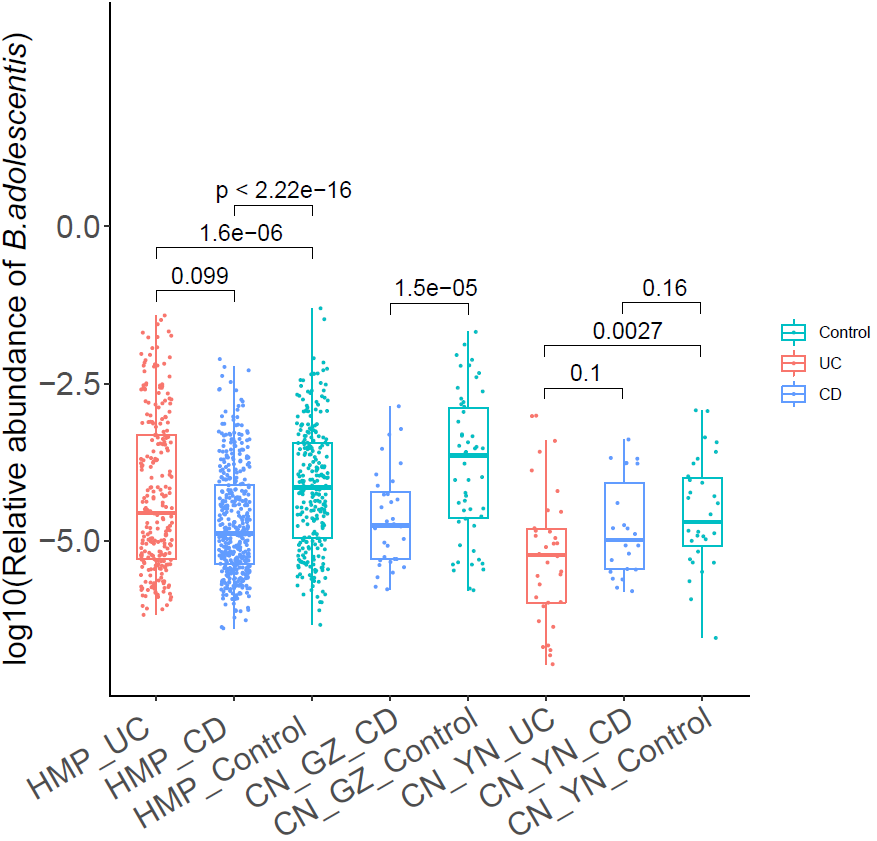


**Figure S1**. Log10 relative abundance of B.*adolescentis* in UCs, CDs, and controls in metagenome cohorts of CN_GZ, CN_YN, and HMP. Mark, wilcoxon test p value.

**Supplementary Table: The Virulence Factors genes.**

| **Strain_gene** | **GeneID** | **Gene** | **VF category:** | **Related VF** |
| --- | --- | --- | --- | --- |
| AF91-08b2A_01010 | VFG018666 | *rfbB* | Immune modulation; Antiphagocytosis | Capsule |
| AF91-08b2A_00247 | VFG001399 | *glnA1* | Nutritional/Metabolic factor | Glutamine synthesis |
| AF91-08b2A_00977 | VFG009376 | *leuD* | Nutritional/Metabolic factor | Leucine synthesi |
| AF91-08b2A_01836 | VFG016493 | *tuf* | Adherence | EF-Tu |
| AF91-08b2A_01833 | VFG043550 | *groEL2* | Adherence | GroEL |
| AF91-08b2A_01794 | VFG006022 | *rfbB* | Immune modulation; Antiphagocytosis | Capsule |
| AF91-08b2A_01803 | VFG006052 | *rfbA* | Immune modulation; Antiphagocytosis | Capsule |
| AF91-08b2A_01834 | VFG006022 | *rfbB* | Immune modulation; Antiphagocytosis | Capsule |
| AF91-08b2A_00756 | VFG047044 | *wbtL* | Immune modulation; Inflammatory signaling pathway | LPS |

**Supplementary Table: Genes associated with antibiotic resistance**

| **ORF_ID** | **Best_Hit_ARO** | **Drug Class** |
| --- | --- | --- |
| AF91-08b2A | *Bifidobacterium* *adolescentis* *rpoB*mutants conferring resistance to rifampicin | rifamycin antibiotic |
| AF91-08b2A | *tet(W)* | tetracycline antibiotic |
| AF91-08b2A | *dfrF* | diaminopyrimidine antibiotic |
| AF91-08b2A | *ErmX* | macrolide antibiotic; lincosamide antibiotic; streptogramin antibiotic;streptogramin A antibiotic; streptogramin B antibiotic |

**Supplementary Table: Important genes encoding probiotic-related genes in Strain AF91-08b2A.**

| **Stress response** | **Gene ID** | **Gene** | **EC** |
| --- | --- | --- | --- |
| Universal stress  family protein | AF91-08b2A_01017 | *uspA* | - |
|  | AF91-08b2A_01115 | *-* | - |
| Heat-shock stress | AF91-08b2A_01520 | *grpE* | - |
|  | AF91-08b2A_01521 | *dnaK* | - |
|  | AF91-08b2A_00349 | *dnaJ* | - |
|  | AF91-08b2A_01519 | *dnaJ* | - |
|  | AF91-08b2A_01648 | *-* | - |
|  | AF91-08b2A_00642 | *groS* | - |
|  | AF91-08b2A_01010 | *groL* | - |
|  | AF91-08b2A_00348 | *hrcA* | - |
| Cold-shock stress | AF91-08b2A_01015 | *cspB* | - |
| Proteases and chaperones | AF91-08b2A_01018 | *clpC* | - |
|  | AF91-08b2A_01032 | *clpP1* | 3.4.21.92 |
|  | AF91-08b2A_01033 | *clpP* | 3.4.21.92 |
|  | AF91-08b2A_01034 | *clpX* | - |
|  | AF91-08b2A_01473 | *clpB* | - |
|  | AF91-08b2A_01018 | *clpC* | - |
| Acid resistance | AF91-08b2A_01441 | *atpC* | - |
|  | AF91-08b2A_01442 | *atpD* | 3.6.3.14 |
|  | AF91-08b2A_01443 | *atpG* | - |
|  | AF91-08b2A_01444 | *atpA* | 3.6.3.14 |
|  | AF91-08b2A_01445 | *atpH* | - |
|  | AF91-08b2A_01446 | *atpF* | - |
|  | AF91-08b2A_01447 | *atpE* | - |
|  | AF91-08b2A_01448 | *atpB* | - |
|  | AF91-08b2A_01036 | *nhaA* | - |
|  | AF91-08b2A_01558 | *nhaP* | - |
| Oxidative stress | AF91-08b2A_01394 | *trxA* | 1.8.1.9 |
|  | AF91-08b2A_01691 | *trxB* | 1.8.1.9 |
|  | AF91-08b2A_01546 | *nfrA* | - |
|  | AF91-08b2A_01547 | *nfrA* | - |
| DNA and protein protection and repair | AF91-08b2A_00544 | *ssb* | - |
|  | AF91-08b2A_00889 | *ssb2* | - |
|  | AF91-08b2A_01287 | *ssb* | - |
|  | AF91-08b2A_01788 | *ssb* | - |
|  | AF91-08b2A_00169 | *recA* | - |
|  | AF91-08b2A_00182 | *gyrB2* | 5.99.1.3 |
|  | AF91-08b2A_00185 | *gyrA2* | 5.99.1.3 |
|  | AF91-08b2A_00379 | *ruvB* | 3.6.4.12 |
|  | AF91-08b2A_00380 | *ruvA* | 3.6.4.12 |
|  | AF91-08b2A_00381 | *ruvC* | 3.1.22.4 |
|  | AF91-08b2A_00483 | *ruvX* | - |
|  | AF91-08b2A_00777 | *topA* | 5.99.1.2 |
|  | AF91-08b2A_00926 | *recQ* | 3.6.4.12 |
|  | AF91-08b2A_00927 | *luxS* | 4.4.1.21 |
|  | AF91-08b2A_01679 | *gyrA* | 5.99.1.3 |
|  | AF91-08b2A_01680 | *gyrB* | 5.99.1.3 |
